# Diversified applications of hepatocellular carcinoma medications: molecular-targeted, immunotherapeutic, and combined approaches

**DOI:** 10.3389/fphar.2024.1422033

**Published:** 2024-09-27

**Authors:** Haoyang Chen, Huihui Liu, Xiaowei Zhang, Suhua Wang, Chunxia Liu, Ke An, Ruijuan Liu, Xin Tian

**Affiliations:** ^1^ Department of Pharmacy, The First Affiliated Hospital of Zhengzhou University, Zhengzhou, China; ^2^ Henan Key Laboratory of Precision Clinical Pharmacy, Zhengzhou, China; ^3^ School of Pharmacy, Zhengzhou University, Zhengzhou, China

**Keywords:** hepatocellular carcinoma, molecular-targeted drugs, sorafenib, immunotherapeutic agents, combination therapies

## Abstract

Hepatocellular carcinoma (HCC) is one of the primary forms of liver cancer and is currently the sixth most prevalent malignancy worldwide. In addition to surgical interventions, effective drug treatment is essential for treating HCC. With an increasing number of therapeutic drugs for liver cancer undergoing clinical studies, the therapeutic strategies for advanced HCC are more diverse than ever, leading to improved prospects for HCC patients. Molecular targeted drugs and immunotherapies have become crucial treatment options for HCC. Treatment programs include single-agent molecular-targeted drugs, immunotherapies, combinations of immunotherapies with molecular-targeted drugs, and dual immune checkpoint inhibitors. However, further exploration is necessary to determine the optimal pharmacological treatment regimens, and the development of new effective drugs is urgently needed. This review provides an overview of the current globally approved drugs for liver cancer, as well as the latest advances in ongoing clinical research and drug therapies. Additionally, the review offers an outlook and discussion on the prospects for the development of drug therapy approaches for HCC.

## Highlights

• Drug treatment remains an essential approach to treating HCC besides surgical interventions, and the therapeutic strategies for advanced HCC are more diversified than before.
• Molecular targeted drugs and immunotherapies used alone or in combination with each other have been crucial therapeutic options for the treatment of HCC.
• The optimal pharmacological treatment regimens for HCC necessitate further exploration, and the new effective drugs need to be developed urgently.

## Introduction

Liver cancer remains a major global health challenge, and its incidence is increasing worldwide (Vogel et al., 2022). It is projected that the annual incidence of new liver cancer cases will rise by over half in the next 10–20 years (Rumgay et al., 2022). Hepatocellular carcinoma (HCC) is the most common form of liver cancer, accounting for the majority of cases (Llovet et al., 2021). Viral hepatitis and cirrhosis are significant risk factors for HCC, with the Hepatitis B virus (HBV) being a more prominent risk factor than Hepatitis C virus (HCV) (Rumgay et al., 2022). HBV can integrate into the host genome, causing direct carcinogenic effects. Even in the absence of cirrhosis, HBV can induce hepatocarcinogenesis through mechanisms such as the activation of oncogenes and the inhibition of tumor suppressor genes (Jiang et al., 2021). HCV promotes HCC through chronic inflammation, oxidative stress, and fibrosis (Guntipalli et al., 2021). The burden of HCC caused by viral hepatitis is substantial. Non-alcoholic fatty liver disease (NAFLD) is also rapidly becoming the primary cause of HCC (Yip et al., 2022). Indeed, HCC is the fourth leading cause of cancer-related death across the world. The highest incidence and mortality of HCC are observed in East Asia and Africa, and these rates are also increasing in different parts of Europe and the USA (McGlynn et al., 2015). According to the Surveillance Epidemiology End Results (SEER), HCC is projected to become the third leading cause of cancer-related death by 2030 (Rahib et al., 2014). This review focuses specifically on the recent advancements in drug therapies for hepatocellular carcinoma (HCC) and their impact on treatment outcomes.

Accurate staging is essential for determining the most appropriate treatment for HCC due to its diverse causes and risk factors. The Barcelona Liver Cancer Clinic (BCLC) staging algorithm, proposed in 1999, is the most widely used system. It classifies patients into five stages (BCLC-0, A, B, C, or D) (Llovet et al., 1999; European Association for the Study of the LiverEuropean Association for the Study of the Liver, 2018). Curative treatment options such as surgical resection, ablation, and liver transplantation are recommended for patients with early-stage liver cancer (single liver tumors or up to 3 nodules ≤ 3 cm), which have been shown to produce the best results with a 5-year survival rate of about 70%–80% (European Association for the Study of the LiverEuropean Association for the Study of the Liver, 2018; Vogel et al., 2018; Marrero et al., 2018; Omata et al., 2017). While surgical resection and liver transplantation are curative options for early-stage HCC, many patients present with advanced disease where systemic therapies play a critical role.

Multiple systemic therapies have been approved in phase III clinical. These include first-line treatments like sorafenib, lenvatinib, atezolizumab plus bevacizumab, and tremelimumab plus durvalumab, as well as second-line treatments like regorafenib, cabozantinib, and ramucirumab (Llovet et al., 2021; Sonbol et al., 2020). On this basis, the U.S. Food and Drug Administration (FDA) has granted accelerated approval of nivolumab, pembrolizumab, and nivolumab plus ipilimumab as second-line treatments. Following systematic evaluation, the atezolizumab and bevacizumab combination is now considered the standard of care for first-line treatment of advanced HCC patients (Sonbol et al., 2020). Moreover, combination therapies involving anti-angiogenesis agents with immune checkpoint inhibitors (ICIs), dual ICIs, and targeted agents in conjunction with surgery or other loco-regional therapies have shown promise and provided the basis for clinical trials (El-Khoueiry et al., 2022).

Despite the availability of several systemic therapies, challenges such as drug resistance and limited efficacy in certain patient populations underscore the urgent need for novel therapeutic approaches. New therapeutic strategies may focus on enhancing treatment efficacy and overcoming drug resistance to expand treatment options, ultimately improving the survival of patients with HCC. This review summarizes the drugs approved for first-line and second-line treatment of HCC in recent years, including their approval dates, targets and mechanisms of action, limitations, and adverse reactions. The purpose is to provide information to support doctors and patients in choosing the best treatment drugs by summarizing the basic situation of existing drugs for liver cancer treatment and to provide directions for researchers to develop drugs with more significant effects and safer use based on current drug foundations.

## Mechanisms of molecular and immunologically targeted therapies

Approved systemic therapies for liver cancer currently focus on targeting molecular receptors or the immune system. The mechanisms underlying the development of liver cancer are complicated and diverse. Preventing hepatitis and fatty liver can somewhat reduce the risk of HCC. However, for patients already diagnosed with cancer, molecular and immune therapies are effective measures.

Molecular targeted therapies, which aim to inhibit molecular pathways critical for tumor growth and maintenance, are the primary treatment approach for advanced HCC.

Vascular endothelial growth factor (VEGF) and platelet-derived growth factor (PDGF) are crucial factors that play significant roles in the neovascularization, invasiveness, and metastatic potential of HCC. The VEGF receptors (VEGFR) are found on endothelial cells and initiate a cascade essential for angiogenesis, making them crucial drivers of tumor vascularization (Mabeta and Steenkamp, 2022). PDGF contributes to angiogenesis by recruiting pericytes and smooth muscle cells to nascent vascular sprouts. The aberrant expression of VEGFR and PDGF receptor (PDGFR) during hepatocarcinogenesis leads to increased angiogenesis formation and activation of the downstream RAS/MAPK pathway, promoting cellular proliferation (Zou et al., 2022; Babina and Turner, 2017). Therefore, drugs that inhibit VEGF and PDGF, such as sorafenib and lenvatinib, are effective therapeutic options for liver cancer (Wilhelm et al., 2008; Zhao et al., 2020).

The fibroblast growth factor (FGF) family comprises at least five fibroblast growth factor receptors (FGFR1-FGFR5) and over 20 homologous ligands. FGFR4 is the predominant receptor found in the human liver, and its endogenous ligand FGF19 is overexpressed to promote HCC survival and enhance its resistance to apoptosis (Yang et al., 2023). FGF8, FGF17, and FGF18 are involved in autocrine and paracrine signaling in hepatocellular carcinoma, augmenting tumor cell survival, angiogenesis, and neovascularization (Xie et al., 2020). These mechanisms underpin the use of FGFR-targeted inhibitors like lenvatinib and regorafenib in treating liver cancer (Zhao et al., 2020; Wilhelm et al., 2011).

MET is a tyrosine kinase receptor for hepatocyte growth factor (HGF). The activation of the HGF-MET pathway is closely associated with the development and progression of HCC. This pathway promotes cell proliferation by regulating cell morphology and motility (Guo et al., 2020). Additionally, elevated expression of HGF-MET correlates with poor survival outcomes in HCC patients (Wang et al., 2020). For HCC with high MET expression, cabozantinib is a therapeutic option. However, selective inhibitors targeting MET specifically are more likely to effectively suppress MET activity while minimizing off-target toxicity, making them the focus of current clinical research (Yang et al., 2023).

The RAS/RAF/MEK/ERK (MAPK) signaling cascade is an important pathway that regulates tumor cell proliferation and differentiation and is closely associated with the metastasis and progression of HCC (Moon and Ro, 2021). The aberrant RAS/MAPK pathway activation is strongly linked to various growth factors, such as epidermal growth factor (EGF), VEGF, PDGF, and HGF (Degirmenci et al., 2020). Consequently, blocking the MAPK signaling pathway represents a potentially effective therapeutic strategy for HCC. Sorafenib and regorafenib are effective inhibitors of the MAPK pathway by targeting RAF, providing a valuable approach in the treatment of liver cancer.

Other receptor tyrosine kinases, including KIT, RET, and AXL, also play crucial roles in developing and progressing HCC (Huang et al., 2020). Aberrant expression of KIT in some HCC patients is associated with specific HCC subtypes, impacting cancer invasiveness and prognosis. Additionally, KIT can influence HCC invasion and metastasis by regulating the expression of genes related to epithelial-mesenchymal transition (EMT) (Pathania et al., 2021). RET, an oncogene-encoded receptor tyrosine kinase, is known for its role in the development and maintenance of the nervous system. However, increasing evidence indicates that RET is also involved in tumor growth and metastasis across various cancer types (Takahashi, 2022). AXL, a TAM receptor tyrosine kinase family member, was initially identified for its role in embryonic development and immune regulation. Nonetheless, its abnormal expression is associated with tumor invasiveness, resistance, and poor prognosis in several cancers, including HCC (Zhu et al., 2019). Cabozantinib, which can target and inhibit KIT, RET, and AXL receptor tyrosine kinases, has been approved as a second-line treatment for HCC that is resistant to sorafenib.

Tumor cells can evade the immune system by hijacking the programmed death-1 (PD-1)/programmed death ligand 1 (PD-L1) pathway or other mechanisms. This aids in tumor development and progression (Yi et al., 2022). To enhance tolerance to harmless foreign molecules, the liver typically maintains an anti-inflammatory environment by expressing and secreting inhibitory molecules (Heymann and Tacke, 2016). Similarly, HCC exhibits an inflammatory yet suppressive immune environment, which is conducive to stronger and more durable responses to immune checkpoint inhibitors. Moreover, the liver contains a substantial number of immune cells, making HCC patients suitable candidates for immunotherapy (Sangro et al., 2021). Several immunotherapy drugs have been approved for first- and second-line treatment of HCC. These drugs primarily target established checkpoints such as PD-1 ligands, PD-L1/2 receptors, and cytotoxic T lymphocyte-associated antigen 4 (CTLA-4) receptors (Ringelhan et al., 2018).

Developing immune-based treatments for HCC has also accelerated the emergence of combination therapies involving immune-related approaches. For instance, dual immune checkpoint inhibitors have proven to be an effective combination therapy. This targets the PD-1/PD-L1 pathway and CTLA-4 receptors, significantly suppressing immune evasion in HCC (Song X. et al., 2023; Yau et al., 2020). Additionally, VEGF inhibitors can create an inflammatory microenvironment that enhances the efficacy of immune checkpoint inhibitors. The use of this combined therapy has demonstrated strong therapeutic effects. It is the mechanism behind the current standard therapy for HCC, atezolizumab plus bevacizumab (Rimini et al., 2022).

**FIGURE 1:**
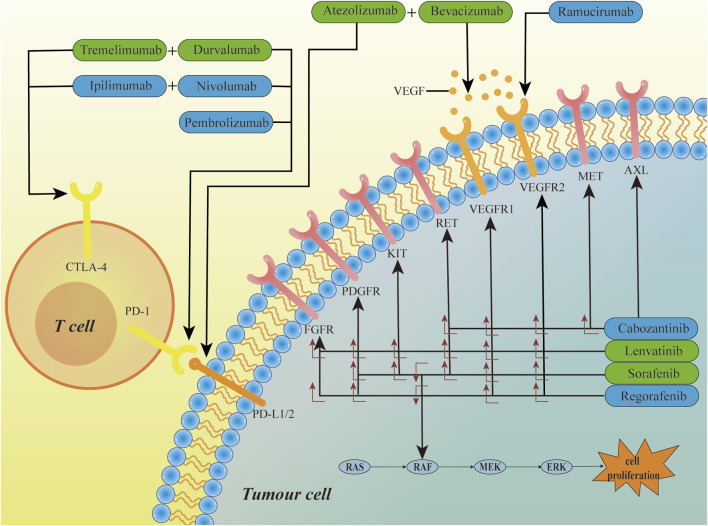
The mechanism of HCC targeted therapy drugs. Sorafenib, lenvatinib, cabozantinib, and regorafenib are small-molecule multi-kinase inhibitors that target multiple kinase receptors, inhibiting tumor growth by blocking biological processes such as angiogenesis. Bevacizumab and Ramucirumab are kinase inhibitors, targeting the VEGF ligand and VEGFR2, respectively. Atezolizumab, Durvalumab, Nivolumab, and Pembrolizumab are immune checkpoint inhibitors that exert their effects by inhibiting the PD-1/PD-L1/2 signaling pathway. Tremelimumab and Ipilimumab exert their effects by inhibiting the CTLA-4 receptors. Green boxes represent first-line treatment drugs, blue boxes represent second-line treatment drugs, and the symbol “+” indicates combination therapy.

Furthermore, we have depicted the targets of drugs currently approved for first-line or second-line treatment of HCC in Figure 1.

## Molecular-targeted monotherapies

Molecular targeted therapy has been a focal point in HCC research. Since the multikinase inhibitor sorafenib was approved for HCC treatment, an increasing number of molecular drugs have shown excellent therapeutic effects in clinical trials for HCC. These include anti-angiogenic drugs such as bevacizumab, tyrosine kinase inhibitors like cabozantinib, and epidermal growth factors inhibitors such as lenvatinib and regorafenib. The application of molecular targeted drugs in liver cancer treatment offers patients more precise, personalized, and effective therapeutic options, with the potential to improve both the quality of life and survival period. However, it is crucial to closely monitor patients’ treatment responses and drug side effects to ensure the safety and efficacy of the treatment. This section focuses on molecular targeted drugs as a standalone therapy, as shown in Table 1.

**TABLE 1:** Summary of molecular-targeted monotherapies for advanced HCC.

Drugs	Classification	Target and mechanism of action	Clinical stage	Study name	Application	MOS (months)	MPFS (months)	TRAES	Refs
Sorafenib	protein kinase inhibitors	RAF, VEGFR1-2, PDGFR, KIT and RET	FDA-approved (2007)	SHARP	First-Line	10.7	4.1	Gastrointestinal, hand-foot skin reactions, abdominal pain, and hypertension	Llovet et al. (2008)
Lenvatinib		VEGFR1-2, FGFR, PDGFR, RET and KIT	FDA-approved (August 2018)	REFLECT	First-Line	13.6	7.4	Liver failure, heart failure, renal failure, hypertensive crisis, and bleeding	FDA, 2018a; Kudo et al. (2018)
Regorafenib		VEGFR1-2, FGFR, PDGFR, RAF, BRAF and KIT	FDA-approved (April 2017)	RESORCE	Second-line	10.6	3.1	Fatigue, hypertension, diarrhea, hand-foot skin reaction, hepatotoxicity, and bleeding	Bruix et al., 2017; FDA (2017a)
Cabozantinib		VEGFR1-2, RET, MET and AXL	FDA-approved (March 2019)	CELESTIAL	Second-line	10.2	5.2	Diarrhea, hypertension, hand-foot skin reaction, bleeding, hypothyroidism, ONJ and RPLS.	Abou-Alfa et al. (2018), FDA (2019a)
Donafenib^a^		VEGFR, PDGFR and RAF	UMPA-approved (January 2021)	ZGDH3	First-Line	12.1	3.7	Hand-foot skin reactions, diarrhea, hypertension, alopecia, rash, decreased appetite	Qin et al. (2021a)
Ramucirumab	monoclonal antibodies	VEGFR2	FDA-approved (May 2019)	REACH-2	Second-line	8.5	2.8	Fatigue, peripheral edema, hypertension, abdominal pain, decreased appetite, proteinuria, ascites, hyponatremia, hypoalbuminemia, and thrombocytopenia	Chau et al., 2017; FDA (2019b)

HCC, Hepatocellular carcinoma; FDA, Food and Drug Administration; MOS, Median overall survival; MPFS, Median progression-free-survival; TRAES, Treatment-related adverse events; ONJ, Osteonecrosis of the jaw; RPLS, Reversible posterior leukoencephalopathy syndrome.

^a^ : Approved by the National Medical Products Administration (NMPA) of China.

### Sorafenib

Sorafenib, the first approved treatment for HCC, owes its efficacy to its inhibition of multiple kinase targets, which promotes cell apoptosis, reduces angiogenesis, and inhibits tumor cell proliferation. Therefore, sorafenib has been the mainstay of treatment for a decade (Tang et al., 2020). The approval of subsequent drugs has primarily been based on comparisons with the efficacy of sorafenib, further underscoring its pivotal role in treating HCC. Sorafenib targets and inhibits several key kinases and receptors involved in cancer cell growth and survival, including RAF kinase, (Liu et al., 2006), VEGFR1-2, PDGFR, KIT, and RET proto-oncogene receptor tyrosine kinase (Wilhelm et al., 2008). It can be used to treat various cancers, including liver, (Llovet et al., 2021), kidney, (Escudier, 2007), and thyroid cancers (Brose et al., 2014). In advanced HCC, sorafenib is currently the effective first-line treatment drug (Tang et al., 2020). It is worth noting that sorafenib is the first targeted therapy drug that has shown efficacy in patients with advanced HCC.

In one key SHARP study, the median overall survival (OS) for HCC patients in the sorafenib group was 10.7 months, while the patients who received a placebo were 7.9 months (Llovet et al., 2008). A parallel Phase 3 study also showed similar results (Ren et al., 2021; Cheng et al., 2009). Although several clinical trials have demonstrated the efficacy of sorafenib in improving OS and progression-free survival in patients with advanced HCC, the reasons for the extension of survival are likely multifactorial, including differences in inclusion criteria and the use of effective sequential therapies (Huang et al., 2020; Cheng et al., 2009). Currently, the clinical benefit of sorafenib is still limited, with only about 30% of patients benefiting from it, and this population usually develops resistance within 6 months (Cheng et al., 2020; Xia et al., 2020; Méndez-Blanco et al., 2018). Sorafenib may cause adverse reactions in some patients, and the frequency and severity of these adverse reactions vary among individuals, mainly including gastrointestinal (diarrhea and weight loss) and hand-foot skin reactions (Rimassa et al., 2019; Li Y. et al., 2015). In severe cases, sorafenib can cause hypertension and abdominal pain, leading to treatment discontinuation (Colagrande et al., 2015; Fu et al., 2018).

However, sorafenib remains an important treatment option for advanced HCC patients, and ongoing research is focused on developing more effective and personalized treatment strategies for this disease. The complex molecular pathogenesis of HCC has stimulated research on combinations of sorafenib with other molecularly targeted drugs. Sorafenib has been combined with antiangiogenic agents, (Morse et al., 2019), MEK/ERK pathway inhibitors, (Huynh et al., 2019), mTOR pathway inhibitors, (Wang C. et al., 2019), histone deacetylase inhibitors, (Chang et al., 2020; Freese et al., 2019) EGF/EGF receptor (EGFR) pathway inhibitors, (Dong et al., 2020), and HGF/c-MET pathway inhibitors (Goyal et al., 2013). Other agents such as interferon, (Tan et al., 2019; Wang M. et al., 2019), selumetinib, (Tai et al., 2016), capecitabine, (Patt et al., 2017), tegafur-uracil, (Azim et al., 2018), gemcitabine and oxaliplatin (GEMOX), (Assenat et al., 2019), and gemcitabine alone have also been evaluated. Still, to date, no treatments involving combinations containing sorafenib have succeeded in phase III trials.

### Lenvatinib

Before lenvatinib was approved for the treatment of HCC, sorafenib has consistently been the preferred first-line treatment drug for HCC. Lenvatinib was approved based on its non-inferiority to sorafenib (Vogel et al., 2021). Lenvatinib is also a multitargeted tyrosine kinase inhibitor (TKI) that targets various receptors, including VEGFR1-2, FGFR, PDGFR, the RET proto-oncogene receptor tyrosine kinase, and KIT proto-oncogene receptor tyrosine kinase (Zhao et al., 2020). By inhibiting these receptors, lenvatinib can help reduce the growth and spread of liver cancer cells. Specifically, it works by blocking the formation of new blood vessels that provide nutrients and oxygen to the tumor, a process known as angiogenesis. Without adequate blood supply, the ability of the tumor to grow and spread is diminished. Lenvatinib can be used to treat various types of cancer, including thyroid, (Ferrari et al., 2021; Fogli et al., 2021), kidney, (Roviello et al., 2018), and liver cancer. The FDA has approved lenvatinib for the first-line treatment of advanced HCC (Yamazaki et al., 2019; FDA, 2018a).

In clinical trials, lenvatinib has been shown to be effective in treating advanced HCC. A parallel Phase 3 study found a statistically significant improvement in OS with lenvatinib compared to placebo in patients with unresectable HCC who had not previously received systemic therapy (Kudo et al., 2018). In the phase 3 REFLECT study, which primarily recruited Asian patients, lenvatinib demonstrated noninferiority to sorafenib in first-line treatment, the median OS in the lenvatinib group was 13.6 months compared to 12.3 months in the sorafenib group (Vogel et al., 2021). For the key secondary endpoints, median progression-free survival, and overall response rate, lenvatinib was superior to sorafenib (Arai et al., 2017). Recently, it has been reported that compared to sorafenib, lenvatinib has demonstrated even better clinical outcomes in the treatment of unresectable hepatitis B virus-related HCC (Choi et al., 2022).

Lenvatinib’s effectiveness against HCC is encouraging, but adverse events during treatment are common and often lead to dose interruption or treatment discontinuation. Common side effects of lenvatinib include fatigue, nausea, diarrhea, hypertension, and decreased appetite. In addition to these common side effects, lenvatinib may cause more severe side effects in some patients, including bleeding or blood clots, liver problems (such as elevated liver enzymes or liver failure), heart problems (such as heart attacks or heart failure), kidney problems (such as renal failure or proteinuria), and hypertensive crisis (Rimassa et al., 2019; Fogli et al., 2021; Arai et al., 2017; Reed et al., 2020; Kim et al., 2022; Nervo et al., 2021). A lower dose that can guarantee efficacy and improve tolerance is being explored, considering also the potential activity of lenvatinib in controlling primary and secondary brain tumors. (Wang R. et al., 2019).

Overall, lenvatinib is considered an important treatment option for advanced HCC patients who are not candidates for surgery or liver transplantation and is typically used as a first-line therapy. However, more research is still needed to explore whether available biomarkers can predict lenvatinib’s efficacy response and resistance to define patient populations better and avoid unnecessary risks (Catalano et al., 2021).

### Regorafenib

Regorafenib is a small molecule inhibitor of multiple kinases, including those involved in angiogenesis and tumor growth (Grothey et al., 2020). Specifically, regorafenib inhibits the activity of VEGFR, PDGFR, and FGFR, all of which are involved in angiogenesis, and other kinases involved in cell proliferation and survival, such as RAF, BRAF and KIT (Wilhelm et al., 2011). Clinical studies have shown that regorafenib is effective in treating HCC patients who have received sorafenib. In a phase III clinical trial, regorafenib was shown to improve OS in HCC patients previously treated with sorafenib significantly. In this study, the median OS of patients receiving regorafenib was 10.6 months compared to 7.8 months for patients receiving the placebo (Bruix et al., 2017). In 2017, regorafenib was approved by the FDA for the treatment of advanced HCC in patients who are intolerant to sorafenib and have progressed on that therapy (FDA, 2017a). The subsequent success of regorafenib in HCC patients who progress on sorafenib treatment heralded a new era of second-line treatment and was quickly followed by ramucirumab, cabozantinib, and the most influential, ICIs. Besides, regorafenib has also been approved for the treatment of metastatic colorectal cancer that has progressed on other therapies, as well as for the treatment of advanced gastrointestinal stromal tumors that are no longer sensitive to imatinib and sunitinib successively (Grothey et al., 2013; Huemer et al., 2020; Demetri et al., 2013). Overall, regorafenib has demonstrated significant efficacy in various types of cancers that are resistant or have progressed after treatment, making it an important second-line therapy for multiple cancers, including HCC (Grothey et al., 2020).

Like all cancer treatments, regorafenib has side effects. Some common side effects of regorafenib in HCC include fatigue, hypertension, diarrhea, and hand-foot skin reaction (also known as palmar-plantar erythrodysesthesia) (McLellan et al., 2015). Additionally, regorafenib can cause more serious side effects in some patients. These include hepatotoxicity (liver damage or failure), bleeding (especially in patients with liver cancer or those previously treated with anticoagulants or antiplatelet agents), and worsening of cardiac issues (especially in patients with a history of heart disease or hypertension). To avoid serious adverse events, a strict treatment plan is necessary before starting regorafenib therapy (Li J. et al., 2015; Maeda et al., 2019).

### Cabozantinib

Cabozantinib is a small molecule inhibitor of multiple tyrosine kinases, including VEGFR, hepatocyte growth factor receptor (HGFR/c-MET), AXL, and RET, cabozantinib works by inhibiting the activity of these tyrosine kinases, resulting in decreased cancer cell growth and proliferation, reduced angiogenesis, and increased cancer cell death (Yakes et al., 2011). Clinical trials have shown that cabozantinib is effective in treating advanced HCC, particularly in patients who have previously received sorafenib (Abou-Alfa et al., 2018). In 2019, cabozantinib was approved by the FDA for the treatment of advanced HCC in patients who have previously received sorafenib (FDA, 2019a). Cabozantinib is also used to treat renal cell carcinoma (RCC), (Tannir et al., 2017) and medullary thyroid carcinoma (MTC) (Brose et al., 2021). As a second-line therapy for HCC, cabozantinib exhibits side effects that are highly similar to those of regorafenib, including diarrhea, hypertension, hand-foot skin reaction, and bleeding (Rimassa et al., 2019; Zuo et al., 2015). Although cabozantinib has less hepatotoxicity than regorafenib, it may cause serious adverse reactions such as hypothyroidism (low thyroid hormone levels), osteonecrosis of the jaw (a rare but serious condition that can cause jaw pain, swelling, and infection), and reversible posterior leukoencephalopathy syndrome (RPLS, a rare and potentially serious neurological disorder that can cause seizures, headaches, and changes in vision) (McGregor et al., 2022). Therefore, cabozantinib is a prescription drug that can only be taken under the guidance and supervision of a healthcare provider.

### Donafenib

Donafenib is a deuterated sorafenib derivative and a novel multikinase inhibitor, including VEGFR, PDGFR, and Raf kinases (Keam and Duggan, 2021). The results of the phase III clinical trial, ZGDH3, have demonstrated that donafenib is superior to sorafenib in improving OS and has good safety and tolerability in Chinese patients with advanced HCC. It is expected to be a potential first-line monotherapy for these patients (Qin et al., 2021a). Based on these results, in June 2021, donafenib was approved for the first time in China for the treatment of uHCC in patients who have not received prior systemic therapy (Administration, 2021).

### Ramucirumab

Ramucirumab is a monoclonal antibody that targets VEGFR-2. By binding to VEGFR-2, ramucirumab blocks signaling pathways that promote tumor angiogenesis, which helps slow cancer cells’ growth and spread (Poole and Vaidya, 2014). Ramucirumab has been approved by the FDA for the treatment of various types of cancer, including gastric or gastroesophageal junction adenocarcinoma, (Fuchs et al., 2019), non-small cell lung cancer, (Arrieta et al., 2017) and colorectal cancer (Debeuckelaere et al., 2019). It is typically used in combination with chemotherapy but may also be used as a single agent in certain cases.

In a phase III clinical trial, patients with advanced HCC treated with ramucirumab had a median OS of 8.5 months and a median progression-free survival (PFS) of 2.8 months, which was better than the group of patients treated with the placebo (Chau et al., 2017). Therefore, ramucirumab was approved by the FDA for the treatment of advanced HCC in 2019, (FDA, 2019b) becoming an effective second-line treatment for HCC that is not a TKI.

## Immunotherapy monotherapies

The normal human liver contains numerous immune cells, but immune suppression has been identified in HCC. This makes immunotherapy a potentially ideal treatment approach for HCC patients, particularly those who experience disease progression with traditional first-line drugs, especially those with severe cirrhosis or cardiovascular diseases and who are intolerant to other TKIs.

Immunotherapeutic drugs approved for HCC treatment mainly target PD1-PDL1/2 or CTLA-4. In this section, we will discuss immunotherapeutic drugs as a standalone therapy for HCC, as shown in Table 2. Additionally, immunotherapy may be associated with specific side effects, necessitating close monitoring of patients’ responses during treatment.

**TABLE 2:** Summary of immunotherapy monotherapies for advanced HCC.

Drugs	Target and mechanism of action	Clinical stage	Study name	Application	MOS (months)	MPFS (months)	TRAES	Refs
Nivolumab^a^	Target: PD-1 Mechanism: immune checkpoint inhibitor	FDA-accelerated approval (September 2017)	CheckMate 459	Second-line	16.4	3.7	Fatigue, rash, pruritus, diarrhea, nausea, asthenia, cough, dyspnea, constipation, decreased appetite, arthralgia, upper respiratory tract infection, and pyrexia	Yau et al., 2020; FDA (2017b), Yau et al. (2022)
Pembrolizumab^a^	Target: PD-1 Mechanism: immune checkpoint inhibitor	FDA-accelerated approval (December 2018)	KEYNOTE 240	Second-line	13.9	3.0	Fatigue, nausea, constipation, dyspnea, vomiting, diarrhea, rash, and acute kidney injury	FDA, 2018b; Zhu et al. (2018), Finn et al. (2020a)

HCC, Hepatocellular carcinoma; FDA, Food and Drug Administration; MOS, Median overall survival; MPFS, Median progression-free-survival; TRAES, Treatment-related adverse events;

^a^ , Drugs granted accelerated approval for second-line treatment of HCC.

### Nivolumab

Nivolumab, marketed as Opdivo, is a PD-1 antibody, and the FDA granted accelerated approval in September 2017 for second-line treatment of advanced HCC patients who are resistant to sorafenib (FDA, 2017b). This is the first FDA-approved immune checkpoint inhibitor for HCC. The efficacy of immunotherapy for HCC is low, but the duration of effectiveness is relatively long. The function of nivolumab is to block the interaction between PD-1 and its ligands, PD-L1 and PD-L2, which are expressed in cancer cells and other cells in the tumor microenvironment (Abedi et al., 2022). By blocking PD-1, nivolumab helps release the immune system to attack cancer cells and shrink tumors.

In the CheckMate-459 trial, nivolumab was compared with sorafenib as a first-line treatment for advanced HCC. Compared to sorafenib, nivolumab did not improve OS in the overall population (Yau et al., 2022). However, nivolumab showed a significant improvement in OS in patients with high PD-L1 expression, a biomarker associated with better response to ICIs.

Overall, nivolumab is an important treatment option for advanced HCC, especially for patients with high PD-L1 expression levels. Ongoing research focuses on identifying biomarkers and combination therapies to improve the response of HCC and other types of cancer to nivolumab and other ICIs.

### Pembrolizumab

Pembrolizumab is also an immune checkpoint inhibitor that targets the PD-1 receptor on immune cells. On 9 November 2018, the US FDA granted accelerated approval of the immunotherapy drug pembrolizumab (Keytruda) for the treatment of HCC (second-line therapy) in patients who are resistant to sorafenib (FDA, 2018b). In terms of clinical data, there is little difference between pembrolizumab and another drug, Opdivo (Zhu et al., 2018).

Recently, in the KEYNOTE-240 trial, the anti-tumor activity and safety of pembrolizumab have been further confirmed. The study showed that compared with placebo, pembrolizumab improved the OS of advanced HCC patients who had previously received sorafenib treatment (Finn et al., 2020a). Similarly, the latest clinical trial has demonstrated that Pembrolizumab can significantly improve OS and PFS in Asian patients (Qin et al., 2023). Overall, pembrolizumab is an important treatment option for advanced HCC, serving as a second-line therapy after sorafenib. Ongoing research is focused on identifying biomarkers and combination therapies to improve the response to pembrolizumab and other ICIs for HCC and other types of cancer.

## Combination therapies

With the increasing resistance of HCC to single drugs, the effectiveness of a single agent becomes more limited. The spotlight is turning toward the combined use of drugs. Combining therapies contributes to better-individualized treatment. Depending on the patient’s immune status, tumor genotype, phenotype, and other characteristics, a suitable combination of immune checkpoint inhibitors and molecular targeted drugs can be selected to maximize treatment effectiveness while minimizing toxic side effects. The most groundbreaking combination involves the use of the immune checkpoint inhibitor atezolizumab and the molecularly targeted drug bevacizumab, which has now become the standard therapy for advanced HCC. Additionally, dual immune checkpoint inhibitors and dual molecular targeted drugs demonstrate promising applications in liver cancer treatment. The relevant content of the combination therapy approach is illustrated in Table 3.

**TABLE 3:** Summary of combination therapies for advanced HCC.

Drugs	Advantages of combination	Clinical stage	Study name	Application	MOS (months)	MPFS (months)	TRAES	Refs
Atezolizumab + bevacizumab	Targeting both angiogenesis and PD-L1 signaling	FDA-approved (May 2020)	IMbrave150	First-Line	19.2	6.8	Hypertension, fatigue, bleeding and proteinuria	Finn et al., 2020b; FDA (2020a)
Tremelimumab + Durvalumab	Targeting PD-1 and CTLA-4 receptors on T cells	FDA-approved (October 2022)	HIMALAYA	First-Line	16.4	3.8	Rash, diarrhea, fatigue, pruritus, musculoskeletal pain, and abdominal pain	FDA (2022b)
Nivolumab^a^ + Ipilimumab^a^	Targeting PD-1 and CTLA-4 receptors on T cells	FDA-accelerated approval (March 2020)	CheckMate040	Second-line	12.5–22.8	—	Diarrhea, fatigue, rash, pruritus, nausea, pyrexia, cough, decreased appetite, vomiting, abdominal pain, decreased weight, hypothyroidism, and dizziness	Yau et al., 2020; FDA (2020b)
Cabozantinib + Atezolizumab	Targets multiple kinase receptors and PD-L1 signaling	Phase III (September 2017)	COSMIC-312	First-Line	15.4	6.8	Diarrhoea, palmar-plantar erythrodysaesthesia, syndrome, decreased appetite, fatigue, hypertension, hypothyroidism	Kelley et al. (2022)
Pembrolizumab + Lenvatinib	Targets multiple kinase receptors and PD-1 receptor	Phase III (December 2018)	LEAP-002	First-Line	21.2	8.2	Hypothyroidism, diarrhoea, palmar-plantar erythrodysesthesia syndrome, decreased appetite, hypertension, fatigue, dysphonia	Llovet et al. (2023)

HCC, Hepatocellular carcinoma; FDA, Food and Drug Administration; MOS, Median overall survival; MPFS, Median progression-free-survival; TRAES, Treatment-related adverse events.

^a^ : Drugs granted accelerated approval for second-line treatment of HCC.

### Atezolizumab plus bevacizumab

Following the approval of sorafenib and lenvatinib, the approval of atezolizumab plus bevacizumab indicates a new era in HCC treatment, where combination therapy is gradually becoming a more prominent treatment choice, replacing monotherapy. Atezolizumab is a monoclonal antibody that targets the PD-L1 protein on cancer cells and disrupts the interaction between PD-L1 and the PD-1 receptor on immune cells. PD-1 is a protein that helps regulate immune responses and prevents them from attacking normal cells in the body. In many types of cancer, including liver cancer, cancer cells can hijack the PD-1 pathway to evade detection and destruction by the immune system (Zhu et al., 2022). This interaction typically prevents immune cells from attacking cancer cells, but by blocking it, atezolizumab can activate the immune system and help it identify and destroy cancer cells (Markham, 2016; Frampton, 2020). Bevacizumab is a monoclonal antibody that targets the VEGF protein. By blocking VEGF, bevacizumab can reduce the blood supply to the tumor and prevent its growth (Garcia et al., 2020). However, due to an increased risk of bleeding with bevacizumab use, endoscopy is required within 6 months before enrollment, and it is strongly recommended that patients with portal hypertension be screened for varices prior to treatment (Cao et al., 2019; Li and Kroetz, 2018; Hsu et al., 2021).

In comparison to sorafenib, the combination of the VEGF-A antibody bevacizumab and the PD-L1 antibody atezolizumab demonstrated an improvement in OS (Ringelhan et al., 2018; Rimini et al., 2022). The IMbrave150 clinical trial, the first positive phase 3 study using an ICI-based regimen, (Galle et al., 2021; Qin et al., 2021b; Finn et al., 2020b), showed the combination therapy significant survival benefits compared to sorafenib. Atezolizumab in combination with bevacizumab was approved by the United States FDA in May 2020 for the treatment of unresectable or metastatic HCC (FDA, 2020a). The combination of atezolizumab and bevacizumab has been recognized by international guidelines as the new standard of care for first-line treatment of advanced HCC (Cheng et al., 2022).

### Tremelimumab plus durvalumab

In recent times, immunotherapy has gained a significant role in the management of cancer. With Atezolizumab plus bevacizumab becoming the new first-line standard therapy for HCC, the combination of various drugs in treating HCC is now being given more attention. As clinical trials progress, dual immune checkpoint inhibitors have shown significant promise in the treatment of HCC. Dual ICIs such as tremelimumab plus durvalumab and nivolumab plus ipilimumab target proteins named PD-1 and CTLA-4 on T cells, respectively, helping to activate them to identify and attack cancer cells (Arru et al., 2021). Tremelimumab and durvalumab were approved by the FDA in the fall of 2022 for the treatment of metastatic non-small cell lung cancer (mNSCLC) in adult patients with sensitizing epidermal growth factor receptor mutations or anaplastic lymphoma kinase genomic tumor aberrations and unresectable HCC (uHCC) in adult patients with the same (FDA, 2022a; FDA, 2022b; Keam, 2023). In addition, a randomized phase III clinical trial named KESTREL showed that tremelimumab and durvalumab in combination displayed durable responses and reduced treatment-related adverse events (TRAEs) in recurrent or metastatic head and neck squamous cell carcinoma (R/M HNSCC), highlighting the promising potential of the combination therapy in multiple cancers (Psyrri et al., 2023).

In a phase III clinical trial named HIMALAYA, tremelimumab plus durvalumab was compared with sorafenib, the standard therapy for advanced HCC. The trial included advanced HCC patients who had not received systemic therapy before. The results showed that the combination therapy improved OS and had manageable safety compared to sorafenib (Song et al., 2023; Kelley et al., 2021). Based on these positive results, the FDA granted breakthrough therapy designation for the combination therapy for the treatment of advanced HCC in 2019, (FDA, 2022b), following the latest standard first-line therapy atezolizumab plus bevacizumab. However, it is worth noting that tremelimumab plus durvalumab is currently limited to adult patients with uHCC (Song et al., 2023; FDA, 2022b).

Currently, the durvalumab monotherapy has not been approved. Some patients have contraindications to ICIs therapy, including those with severe autoimmune diseases and those who have undergone major organ transplants (Wichelmann et al., 2021). For these patients, monotherapy with sorafenib or lenvatinib is an appropriate first-line therapy.

### Nivolumab plus ipilimumab

The mechanism of action of nivolumab and ipilimumab is to enhance the immune system’s ability to attack cancer cells. By blocking the PD-1 receptor, nivolumab enables T cells to more effectively recognize and attack cancer cells. On the other hand, CTLA-4 is a protein that inhibits the immune system’s response. By blocking CTLA-4 with ipilimumab, the immune system’s response to cancer cells is enhanced. The combination of these two drugs has been demonstrated to effectively treat liver cancer, especially HCC.

Multiple clinical trials have studied the combination of nivolumab and ipilimumab for the treatment of HCC. In a recent phase II trial, the combination of nivolumab and ipilimumab was compared to sorafenib, the standard of care for advanced HCC (Yau et al., 2020). The study showed that the combination therapy significantly improved OS compared to sorafenib. It is important to emphasize that not all patients are suitable for this treatment.

In March 2020, the FDA granted accelerated approval for the use of the PD-1 inhibitor nivolumab in combination with the CTLA-4 inhibitor ipilimumab for the treatment of patients with HCC who have previously received sorafenib treatment (FDA, 2020b). Nivolumab plus ipilimumab is the first dual ICI therapy approved by the FDA for patients with HCC, and is the most effective immunotherapy regimen, even for lung cancer, (Hellmann et al., 2019) kidney cancer, (Motzer et al., 2018), and colon cancer, (Overman et al., 2018) with outstanding efficacy.

### Cabozantinib plus atezolizumab

Currently, cabozantinib plus atezolizumab is being investigated as an emerging combination therapy for the treatment of advanced HCC (Esteban-Fabró et al., 2022). In a phase III clinical trial named COSMIC-312, previously untreated advanced HCC patients were randomly assigned to receive cabozantinib plus atezolizumab or sorafenib, the standard treatment for advanced HCC (Kelley et al., 2022). The study found that combination therapy was associated with improved OS and progression-free survival compared to sorafenib. Based on these hopeful results, the FDA has granted breakthrough therapy designation for the use of cabozantinib plus atezolizumab to treat advanced HCC. This designation aims to accelerate the development and review of new therapies for severe or life-threatening diseases. However, further evaluation of the efficacy of cabozantinib plus atezolizumab in the treatment of advanced HCC is needed. In addition, the combination of cabozantinib and atezolizumab has shown promising results in the treatment of advanced RCC for patients who have previously received anti-angiogenic therapy (Pal et al., 2021).

### Pembrolizumab plus lenvatinib

To improve the response to pembrolizumab or other ICIs for HCC. Combination therapies have become an essential choice in the treatment of HCC. Recently, in the KEYNOTE-524 trial, pembrolizumab was also studied as a first-line treatment for advanced HCC (Finn et al., 2020c). In this study, pembrolizumab was combined with the targeted therapy drug lenvatinib and compared with lenvatinib alone. The study found that the combination of pembrolizumab and lenvatinib improved progression-free survival and OS compared to lenvatinib alone (Sun et al., 2022).

Pembrolizumab showing promise in combination with lenvatinib as a new first-line treatment approach (Rizzo et al., 2022). To further evaluate the safety and efficacy of the pembrolizumab plus lenvatinib therapy, a phase III clinical trial named LEAP-002 is currently underway, lenvatinib plus pembrolizumab has promising antitumor activity in uHCC (Llovet et al., 2023).

## Future perspectives

With the continuous in-depth research on liver cancer, an increasing number of emerging therapeutic methods and drugs are being discovered. Despite multiple first- and second-line regimens being approved for systemic treatment of HCC, the development of resistance and limitations of existing drug therapies for HCC remind us that we cannot stop researching HCC and the urgent need for more precise drug selection, the discovery, and development of drugs with more significant efficacy and safer usage to curb the incidence and progression of HCC in the population. A crucial aspect is the identification and discovery of biomarkers for drug treatment. For instance, mutations in the PI3K-AKT-mTOR pathway serve as genomic biomarkers for sorafenib treatment, while serum biomarkers like angiopoietin-2 (ANG2) and fibroblast growth factor 21 (FGF21) are associated with lenvatinib treatment (Choi et al., 2022; Song et al., 2023). In the realm of immunotherapy, in addition to the established PD-1 and PD-L1, there is potential for CD3 and CD8 to serve as biomarkers (Duffy et al., 2017; Sangro et al., 2020). Biomarkers facilitate personalized treatment, contributing to the specificity and efficacy of drug therapies. Detecting biomarkers enables the monitoring of tumor progression, facilitating adjustments in treatment plans. Furthermore, biomarkers are crucial in new drug development, aiding in assessing drug safety and efficacy.

Immunotherapy, as an emerging and effective cancer solution, holds tremendous potential. Expanding current immunotherapies in drug treatment for HCC is of significant importance. Different from traditional immunotherapeutic drugs, adoptive cell therapy (ACT) enhances the anti-tumor immune response by injecting immune cells, making it more suitable for solid tumors (Roddy et al., 2022). Therefore, ACT is considered feasible in treating liver cancer, and related clinical trials have shown promising results (Shi et al., 2020). Peptide vaccines can induce T-cell responses, enhancing specific anti-tumor immunity and represent a hopeful immunotherapeutic approach for HCC (Mizukoshi et al., 2015).

In addition to general combination therapies, sequential combination treatment is an emerging therapeutic approach that can maintain the synergistic effects of drugs while reducing the combined toxicity. Another similar strategy is alternate treatment, primarily targeting tumor sensitivity to one drug at the cost of developing resistance to another. However, the effectiveness of this approach in HCC is still under debate.

An iterative approach to targeted drugs has provided a wealth of data and encouraging results, particularly in those patients where resistance to sorafenib develops. The understanding and management of HCC have changed significantly due to extensive basic and clinical research over the last decade. However, HCC remains a devastating disease with a widespread and enormous impact on healthcare systems worldwide. In recent years, there have been significant advances in understanding the mechanisms that lead to rapid progress in treating patients with cancer and liver diseases. This collaborative effort involves oncologists, hepatologists, and basic scientists, and it provides hope for continued improvement in patient prognosis.

## Glossary

HCC: Hepatocellular carcinoma
HBV: Hepatitis B virus
HCV: Hepatitis C virus
NAFLD: Non-alcoholic fatty liver disease
SEER: Surveillance Epidemiology End Results
BCLC: Barcelona Liver Cancer Clinic
FDA: Food and Drug Administration
ICIs: Immune checkpoint inhibitors
VEGF: Vascular endothelial growth factor
VEGFR: Vascular endothelial growth factor receptor
PDGF: Platelet-derived growth factor
PDGFR: Platelet-derived growth factor receptor
FGF: Fibroblast growth factor
FGFR: Fibroblast growth factor receptor
HGF: Hepatocyte growth factor
RET: Rearranged during transfection
PD-1: Programmed death-1
PD-L1: Programmed death ligand 1
CTLA-4: Cytotoxic T-lymphocyte-associated antigen 4
OS: Overall survival
EGF: Epidermal growth factor
EGFR: Epidermal growth factor receptor
GEMOX: Gemcitabine and oxaliplatin
TKI: Tyrosine kinase inhibitor
mNSCLC: Metastatic non-small cell lung cancer
uHCC: Unresectable HCC
TRAEs: Treatment-related adverse events
R/M HNSCC: Recurrent or metastatic head and neck squamous cell carcinoma
HGFR: Hepatocyte growth factor receptor
RCC: Renal cell carcinoma
MTC: Medullary thyroid carcinoma
RPLS: Reversible posterior leukoencephalopathy syndrome
PFS: Progression-free-survival
METIV-HCC: High MET expressing HCC
ANG2: Angiopoietin-2
FGF21: Fibroblast growth factor 21
ACT: Adoptive cell therapy
